# Application of Chitosan and Pectin‐Based Nanoemulsion Coating for the Preservation of Plum (*Prunus domestica*) Fruit

**DOI:** 10.1002/fsn3.70824

**Published:** 2025-09-04

**Authors:** Shahzad Zafar Iqbal, Muhammad Waseem, Farah Naz, Munawar Iqbal, Muhammad Adnan Ayub, Osama A. Mohammed, Ahmad Faizal Abdull Razis

**Affiliations:** ^1^ Food Safety and Toxicology Lab, Department of Applied Chemistry Government College University Faisalabad Faisalabad Pakistan; ^2^ School of Energy and Environment City University of Hong Kong Kowloon Tong, Hong Kong China; ^3^ School of Chemistry University of Punjab Lahore Pakistan; ^4^ Department of Chemistry University of Sahiwal Sahiwal Pakistan; ^5^ Department of Pharmacology, College of Medicine University of Bisha Bisha Saudi Arabia; ^6^ Department of Food Science, Faculty of Food Science and Technology Natural Medicines and Products Research Laboratory, Institute of Bioscience (IBS) Laboratory of Food Safety and Food Integrity, Institute of Tropical Agriculture and Food Security (ITAFoS) Universiti Putra Malaysia Selangor Serdang Malaysia

**Keywords:** edible coating, food packaging, food preservation, food safety, plum fruit

## Abstract

Pectin, a bioactive polysaccharide, was mixed with chitosan (CS) and blended with three essential oil formulations to prepare nanoemulsion‐based edible coatings. Three nanoemulsion‐based coatings, C_1_, C_2_, and C_3_, comprising chitosan and pectin at ratios of 1:1, 1.25:1.25, and 1.50:1.50, respectively, were applied to plum fruits and stored at 7°C for 18 days to evaluate their preservation effectiveness. The results have documented the stability and compatibility of prepared nanoemulsions when applied during the fruit storage period. The nanoemulsion C_3_ inhibited *E. coli* and *S. aureus* by 1.20 ± 0.06 and 0.90 ± 0.09 mm, respectively. Furthermore, the C_3_ coating effectively delayed key quality deterioration parameters (*p* < 0.05), such as reduced weight loss (7.75%), maintained firmness (2.50 kg/cm^2^), lower decay incidence (2.80%), respiration rate (RR, 6.80 CO_2_/kg/h), and ethylene production (3.80 μL/kg/h). It also preserved titratable acidity (1.77%), total soluble solids (8.30°Brix), total phenolic content (160 mg GAE/100 g FW), pH values (3.35), and overall acceptability (6.60 score). These results established a robust foundation for the development and application of the active edible coating for prepared nanoemulsions C_3_, C_2_, and C_1_ to preserve postharvest quality and prolong the shelf life of fruits and vegetables.

## Graphical abstract

Pectin‐chitosan‐based edible coating for fruits. The characterization showed good results of the prepared coatings. The application of three coatings has shown a marked effect on the shelf life of plum fruit.

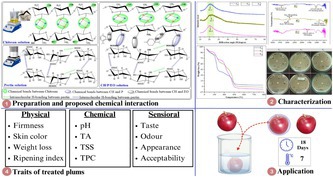

## 1 Introduction

China is the largest plum producer, producing 6,775,221 tons in 2022, followed by Romania, with 665,730 tons, and Pakistan, with 59,676 tons (World Population Review 2024). The global trade in the food sector has increased the accessibility of fresh agricultural products worldwide, enabling year‐round availability of fruits and vegetables (Borsellino et al. 2020). This expanded reach necessitates the delivery of fresh food over long distances, often across multiple territories, to reach consumers in various locations. To withstand the protracted transportation from fields to markets and to provide prolonged storage with quality preservation, preventive measures are crucial in protecting fresh products from potential damage and contamination (Zahra et al. 2025). Postharvest losses significantly impact food products because of their perishable characteristics, representing 28%–55% of the annual total production loss in developing countries, amounting to approximately USD 750 billion annually, and posing a serious threat to food security (Karoney et al. 2024).

Recent advancements in the synthesis and application of edible coatings hold promise for the horticulture and food industries, enabling the prolongation of perishable product shelf life while preserving their intrinsic qualities (Chettri et al. 2023). Utilizing edible coatings on food surfaces can extend the shelf life of products without significantly altering their inherent properties (Miteluț et al. 2021). These coatings may contain active compounds that confer specific functional advantages to food products, including antibacterial, antioxidant, biocatalytic, or nutraceutical properties (Manzoor et al. 2023; Barbosa et al. 2022; Peerzada et al. 2023). Chitosan, a cationic linear amino polysaccharide (poly β‐(1,4) N‐acetyl‐D‐glucosamin), is a bioactive polymer that was obtained by deacetylation of chitin in the presence of an alkali medium (Bisht et al. 2022). Chitosan creates a semi‐permeable layer on the surface of the fruit skin, which reduces or suppresses the atmosphere around the fruit surface and slows down the ripening process (Dhall 2013). The edible coating on the basis of CS exhibited excellent mechanical properties because of its resistance to exchanging gases, such as CO_2_, O_2_, and ethylene (Elsabee and Abdou 2013). Furthermore, it exhibits excellent antioxidant properties (Nair et al. 2018). Pectin, a key component of the cell wall composed of α 1,4‐linked polygalacturonic acids, is an amorphous colloidal carbohydrate deemed safe by the Food and Drug Administration (Rohasmizah and Azizah 2022). Pectin is crucial in food production as a stabilizer, thickening agent, and gelling agent in beverages, jams, yogurt, fruit‐flavored milk drinks, and ice cream (Padmaja et al. 2015). Chitosan and pectin play essential roles in fruit coating applications. Generally, chitosan acts as an antimicrobial and film‐forming agent, and because of these features, it creates a semipermeable barrier that reduces moisture loss and gaseous exchange, subsequently lowering microbial growth and fruit ripening (Dai et al. 2025). Pectin, a biopolymer famous for its gelling properties, enhances the mechanical strength and flexibility of the coating on the fruit surface, helping to retain fruit firmness and protect against oxidative degradation. Plum fruits, which are climacteric fruits, are prone to rapid softening, microbial decay, and moisture loss. These polymers, together, protect the plum fruit by providing a protective layer and extending its shelf life.

Previously, Abdalla et al. (2023) have investigated CS‐pectin and essential oil‐based nanoemulsion for the preservation of strawberries and documented that the strawberries were preserved for 14th day storage period; however, sensory evaluation, which is vital for evaluating taste, quality, smell, and overall acceptability, was not performed (Abdalla et al. 2023). Chu et al. (2025) have fabricated a CS‐pectin‐based nanoemulsion and documented its stability for use as an ultraviolet filter, specifically for non‐food packaging applications. However, numerous studies have been conducted on the separate applications of CS or pectin.

The present study involved the amalgamation of CS, a cationic polymer, with pectin, an anionic bioactive polymer, incorporating optimal concentrations of essential oils (cinnamon, peppermint, and rosemary). The developed nanoemulsion coatings were utilized to preserve plum fruit for 18 days at a temperature of 7°C. In current research, both bioactive polymers are used to evaluate their efficacy and application in maintaining the shelf life of fruits. Furthermore, the characterization of the prepared nanoemulsions using analytical techniques, including particle size, zeta potential, Fourier transform infrared (FTIR), X‐ray diffraction (XRD), and thermogravimetric analysis (TGA), as well as their antibacterial activities, was documented. The developed nanoemulsions demonstrate potential for application in the food industry for edible packaging to preserve quality and safety.

## 2 Materials and Methods

### 2.1 Materials

CS (93% acetylation and molecular weight of 175.16 g mol^−1^), pectin (62%–66% esterification), acetic acid (99.7%), Tween 80 (polysorbate), and essential oils (EOs), including cinnamon, peppermint, and rosemary (100% pure), were purchased from the commercial market (Chiltan Pure, Lahore, Paksiatn). Sodium hydroxide (Sigma‐Aldrich, Darmstadt, Germany) and deionized water were used in this experimental work. Plum fruits were purchased from the local markets of Faisalabad, Punjab, Pakistan. The fresh fruit was selected on the basis of its consistent size, shape, color, and ripeness. Furthermore, the fruit was free from damage or any spoilage.

### 2.2 Preparation of CS‐Pectin Essential Oil‐Based Coating

The edible coating was made using the technique described by Iqbal, Haider, et al. (2024) and consisted of a nanoemulsion of CS, pectin, and EOs, with some modifications. Several concentrations of CS and pectin were prepared and tested (1:1, 1:2, 1:3, 1:4, and 2:1). The coating with a 1:1 ratio of CS and pectin exhibited the highest stability among all the formulations tested. Consequently, this composition was employed to prepare all subsequent coatings. To prepare three distinct nanoemulsion formulations, CS and pectin were first dissolved in a mixture with constant magnetic stirring at 1200 rpm for 1 h to obtain a homogeneous mixture. The stabilized mixtures labeled C_1_, C_2_, and C_3_ (listed in Table 1) were selected for their optimal formulations. Table 1 shows that solutions with different concentrations were prepared, utilizing Tween 80 as an emulsifier and glycerol as a plasticizer. The resulting mixtures were then homogenized at 8000 rpm for 15 min using a high‐pressure homogenizer (T25 digital Ultra‐Turrax; IKA, Staufen, Germany) to ensure uniform dispersion. The oil mixtures were incorporated into the prepared solutions in the final phase to create a coarse emulsion. This emulsion was then sonicated for 1 h using an ultrasonication probe machine (VCX 500; Vibra‐Cell, Newtown, CT, USA) to produce an oil‐embedded nanoemulsion. The solution was subsequently cooled in an ice bath at 20°C while stirring, rested for 60 min, and then stored in a refrigerator.

**TABLE 1:** Combinations of CS and pectin‐based nanoemulsion for the coating of plum fruit.

NE code	CS (*w/v*)	Pectin (*w/v*)	Cinnamon, peppermint, & rosemary mL (v/v/v)	Tween 80 (*w/v*)	Glycerol (*w/v*)
C_1_	1	1	1:1:1	3.5	0.6
C_2_	1.25	1.25	2:1:1	3.5	0.6
C_3_	1.50	1.50	2:2:1	3.5	0.6

### 2.3 Treatments of Plum ( *Prunus domestica* ) Fruits

A total of 50 plum fruit samples were collected, rinsed with tap water, and allowed to drain for 10 min. The chosen plum fruits were randomly allocated into four groups: control group (C_0_), C_1_, C_2_, and C_3_ coated samples. In each group, there were five samples, and the plum fruit samples were immersed in the prepared coating formulations for 10 s and then air‐dried for 15 min at room temperature (27°C); each batch was executed three times to ascertain the anticipated thickness of the coating layer on the basis of early results (Vitti et al. 2024). The samples were kept in polypropylene trays measuring 14 × 9 × 7 cm^3^ at a storage temperature of 7°C ± 0.50°C for 18 days. Throughout the storage period, measurements of physical, physiological, and biochemical characteristics were taken every 3 days.

### 2.4 Characterization of Prepared Nanoemulsion

#### 2.4.1 Particle Size, Particle Dispersion Index (PDI), and Zeta Potential of Nanoemulsions

The particle size, PDI, and zeta potential of prepared nanoemulsions were analyzed using a Zeta sizer (Malvern Panalytical, Worcestershire, UK). The measurements were conducted on aqueous samples diluted at a 2:1 ratio. The studies were performed in triplicate under controlled conditions, with the temperature kept constant at 25°C (Fontes‐Candia et al. 2020).

#### 2.4.2 Attenuated Total Reflectance (ATR)‐FTIR

Nanoemulsion samples were placed on the attenuated total reflectance (ATR) crystal, and maximum pressure was applied with a clamp to ensure close contact between the sample and the ATR crystal. ATR‐FTIR spectra were recorded using an Agilent Cary 630 Fourier transform infrared (FTIR) spectrometer (Agilent, Waldbronn, Germany) with a diamond ATR module. The spectra were captured within the 4000–400 cm^−1^ range, at a resolution of 4 cm^−1^, with a minimum of 32 scans averaged (Asdagh et al. 2021).

#### 2.4.3 X‐Ray Diffraction (XRD)

The crystalline structure of the nanoemulsions was analyzed using an X‐ray diffractometer (XRD D8ADVANCE; Bruker, Dresden, Germany), operating at 42 kV, 30 mA, and 1.54 Å. Spectra were recorded with CuKα radiation, and diffraction patterns were collected at 2*θ* angles ranging from 5° to 60° at room temperature (27°C) (Chen et al. 2023).

#### 2.4.4 Scanning Electron Microscopy (SEM)

The freeze‐dried CS/pectin‐based surface morphology and the essential oil‐loaded nanoemulsion were evaluated using high‐resolution scanning electron microscopy (HR‐SEM). The analysis utilized an Evo‐18 microscope (Zeiss, Oberkochen, Germany) with an operating voltage of 15 kV. Before imaging, the samples were coated with a thin layer of gold using a Nova NanoSEM 250 sputter coater (Quorum, Q150R‐ES; Lewes, East Sussex, UK) to enhance their electrical conductivity.

#### 2.4.5 TGA

TGA was conducted to assess the thermal stability of CS/pectin‐EOs‐based nanoemulsions (Shimadzu, THA‐50, Kyoto, Japan). The emulsion samples were cooled from room temperature to −85°C, leading to water freezing in the bulk. TGA was conducted with an oxygen flow rate of 50 mL/min, from −85°C to 150°C, and a heating rate of 10°C/min (Du et al. 2024).

#### 2.4.6 Antimicrobial Activity Evaluation

The antibacterial activity of the nanoemulsion was assessed using the agar disc diffusion method, following the procedure outlined by Du et al. (2024). The study targeted *Staphylococcus aureus* (ATCC25923) and *Escherichia coli* (MG1655) as Gram‐positive and Gram‐negative, respectively. The bacterial strains were purchased from the American Type Culture Collection (ATCC; Rockville, MD, USA) and maintained in the Industrial Biotechnology Lab, Department of Biochemistry, Government College University Faisalabad. Bacterial suspensions were prepared by mixing 0.30 mL of an overnight culture with 8 mL of saline solution containing 0.85% NaCl, following the 0.50 McFarland standard. The prepared nanoemulsion was dispersed on the agar plate by evenly dispersing it on the surface. The nanoemulsion was carefully pipetted onto the plate and spread uniformly to ensure consistent coverage. Circular discs, 1.70 cm in diameter, were coated with the nanoemulsion and sterilized under UV radiation for 20 min in a laminar airflow environment.

### 2.5 Qualitative Attributes of Plum

#### 2.5.1 Weight Loss, Firmness, and Decay Incidence of Plum

Plums were weighed at the beginning and at regular intervals throughout the storage period. Weight loss (WL) was calculated as a percentage of the initial total weight using the following formula:

(1) $$\;\text{Weight loss}\;\left(\%\right)=\frac{w_{0}-w_{t}}{w_{0}}\times 100$$

where *w*_0_ is the initial weight of plum fruit (g) and *w*_*t*_ is the weight during the storage period of 18 days.

The firmness of plum fruits was evaluated using a modified version of the initial method (Panahirad et al. 2020). A texture analyzer (TA.XT.plus; Stable Micro System Co. Ltd., UK) equipped with a 2 mm diameter p/2 probe was used to assess the firmness of plum fruits. The pre‐test, test, and post‐test speeds were set at 1, 0.50, and 1 mm s^−1^, respectively. The probe reached a depth of 5 mm through the apple. Three sites were selected for study along the cross‐section of each sample, and five fruits were picked randomly for each treatment group.

The number of decayed fruits was recorded during 0, 3, 6, 9, 12, 15, and 18 days of storage. The decay rate was calculated using the following formula:

(2) $$\;\text{Decay rate}\;\left(\%\right)=\frac{\;N_{t}}{N_{0}}\;\times 100$$

Here, *N*_0_ represents the initial number of plum fruits, and *N*_*t*_ is the number of rotten fruits during 0, 3, 6, 9, 12, 15, and 18 days of storage.

#### 2.5.2 Respiration Rate and Ethylene Production

Plumb fruits' RR was measured using our previous method, Iqbal, Hussain, et al. (2024), with minor changes. Four batches (one without coating and three with coatings) were weighed and placed in a tightly sealed, temperature‐controlled container for each treatment. A CO_2_ sensor (Testo‐AG‐435‐2; Germany) was inserted to assess the CO_2_ concentration at one‐minute intervals over 60 min. The RR results were reported as milligrams of CO_2_/kg/h.

Ethylene production was measured by drawing a 1 mL gas sample from the chamber using a syringe and injecting it into a gas chromatograph (QP2010 Ultra; Shimadzu, Japan) designed with a flame ionization detector and a capillary column (RTX‐5; USA). The results were presented as μL/g/h (da Costa de Quadros et al. 2020).

#### 2.5.3 Color Indices

The plums' color was evaluated using a Minolta CR‐400 colorimeter (Konica Minolta Sensing Inc., Osaka, Japan) on 0, 3, 6, 9, 12, 15, and 18 days of storage period. The mean values of a* (red‐green axis), L* (lightness), and b* (yellow‐blue axis) were measured (Prakash et al. 2020).

#### 2.5.4 Titratable Acidity (TA) and Total Soluble Solids (TSS)

TA of control and coated plum fruits was assessed using a standard titration method. The sample, 10 g of plums, was mixed in 100 mL of deionized water and filtered through cotton wool. Then 10 mL of the filtrate was titrated with 0.1 mol/L NaOH until the pH reached 8.0 with phenolphthalein as an indicator. The experiment was repeated three times to achieve average readings.

The TSS content, measured in degrees Brix (°Brix), was evaluated using a digital refractometer (model PR1; Atago Co. Ltd., Japan) at room temperature (27°C). A drop of plum juice was placed on the refractometer to assess the refractive index. Each treatment was conducted in triplicate, and average values were recorded on days 0, 3, 6, 9, 12, 15, and 18 of the storage period (Iqbal et al. 2025).

#### 2.5.5 Total Phenolic Content (TPC) and pH

Folin–Ciocalteu (FC) reagent method was used for the detection of TPC (da Costa de Quadros et al. 2020). A 0.50 mL sample of plum juice was diluted with distilled water to a total volume of 10 mL, from which a 0.10 mL sample was taken. This was mixed with 1.50 mL of prepared FC reagent (10 mL FC: 90 mL distilled water) and 4 mL Na_2_CO_3_. The final volume was maintained at 10 mL with the addition of distilled water and left for 30 min in the dark. Absorbance was measured at 650 nm using a spectrophotometer (Spectronic 20D+; Thermo Scientific, Massachusetts, USA), and the TPC was expressed as mg of gallic acid equivalents per 100 g of fresh weight (FW).

To determine the pH, 10 g of plum fruit samples were mixed with 100 mL of distilled water and stirred for 20 min. After homogenization, the pH was measured using a pH meter (HANNA HI 9318; Leighton Buzzard, Bedfordshire, UK).

### 2.6 Sensory Evaluation

Sensory evaluations were analyzed during storage periods 1, 3, 6, 9, 12, 15, and 18 days. Ten semi‐trained judges assessed the control and coated samples of plum samples for appearance, taste, firmness, odor, color, and overall acceptance at room temperature (27°C). A nine‐point descriptive hedonic scale assessed overall enjoyment, with 9 indicating a strong preference and 1 indicating a strong aversion. The data were adjusted to the average score assigned to each attribute by each panelist. A score of 3.5 was established as the minimum acceptable level.

The study was reviewed and approved by the University Ethical Committee Review Board under approval # GCUF/70/23‐12‐24, and informed consent was obtained from each subject prior to their participation in the study.

### 2.7 Statistical Analysis

Each experiment was conducted with three replicates, and the data analysis was conducted using a one‐way analysis of variance (ANOVA) to assess and compare each treatment efficacy at specific time intervals (3‐day interval). Variance was assessed with Origin Pro software. Duncan's test was performed to compare mean values across various storage intervals. Statistical significance was determined at (*p* < 0.05).

## 3 Results and Discussion

### 3.1 Particle Size, PDI, and Zeta Potential

**FIGURE 1:**
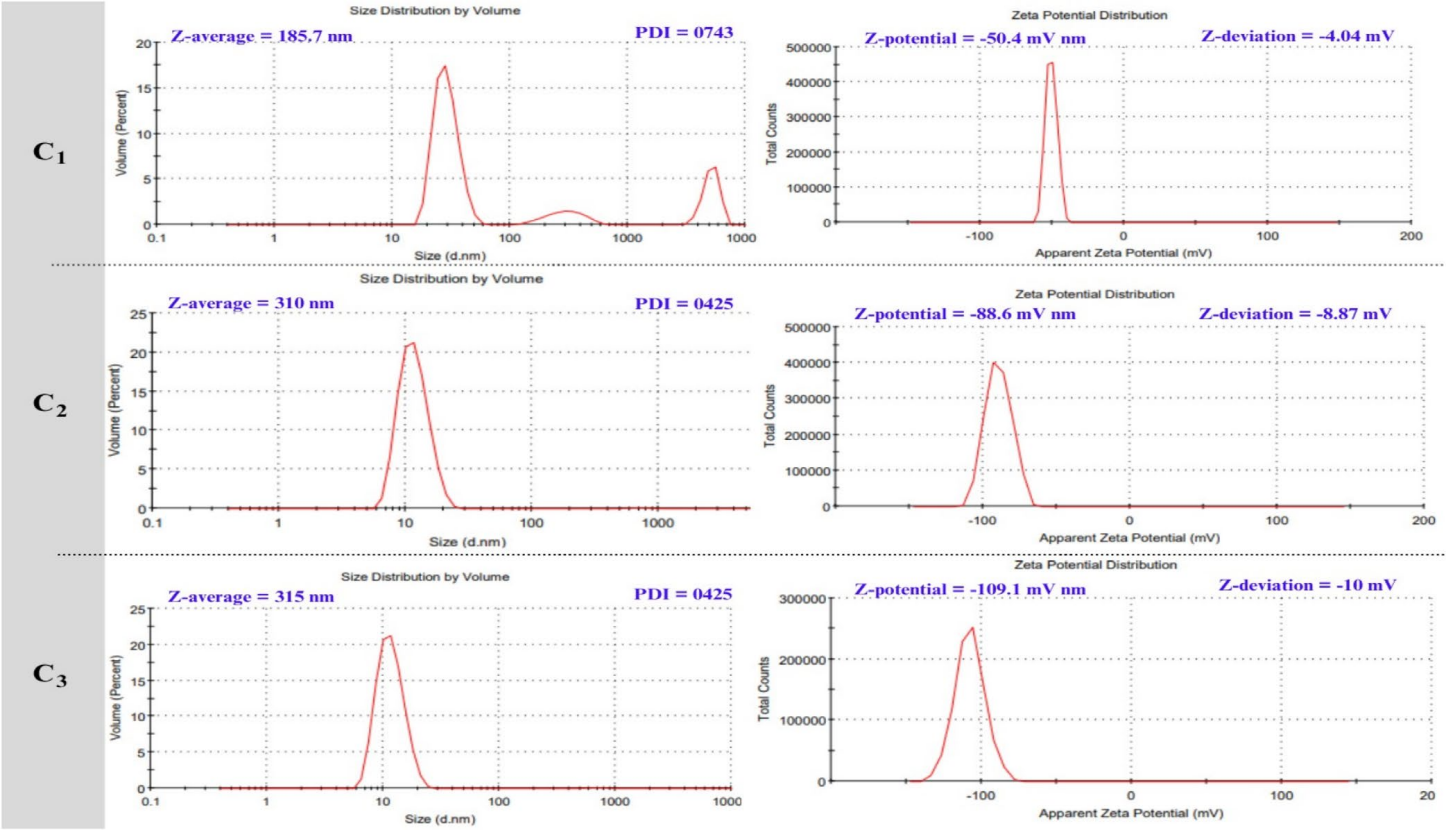
Zeta sizer and zeta potential analysis of prepared nanoemulsions.

The average particle size, PDI, and zeta potential of the prepared nanoemulsions (containing CS‐pectin/EOs) were analyzed to assess the uniformity, stability, and surface charge (Figure 1). The average particle sizes for the C_1_, C_2_, and C_3_ nanoemulsions were 185.70, 310, and 315 nm, respectively. The results indicated a significant increase in particle size (*p* < 0.05) as the concentration of EOs increased from 1% to 3%. Higher concentrations of the CS‐pectin blend exhibited a trend of increasing particle size, indicating that higher polymer concentrations result in the formation of larger particles because of increased solution viscosity, which delays droplet breakdown during emulsification. The larger particles in the C_3_ sample may help maintain the nanoemulsion's stability by increasing its thickness, blocking steric flow, and enhancing electrostatic repulsion (Li et al. 2024).

The PDI values, which provide insight into the distribution of particle sizes within the samples, revealed a broad distribution for C_1_ with a PDI of 0.74. In contrast, the C2 and C3 nanoemulsions exhibited lower PDI values of 0.42, indicating a uniform particle size distribution. The results suggest that PDI values below 0.50 indicate good homogeneity, and therefore, nanoemulsions C2 and C3 exhibit better stability than nanoemulsion C1 (Azadi et al. 2023). The nanoemulsion‐based coatings exhibit superior efficacy in preserving fruit quality during storage as compared to control treatments or conventional coatings. For instance, the nano‐sized droplets in the emulsion system provide a uniform and compact coating layer, which generally enhances the adhesion to the surface of fruit and subsequently delays fruit ripeness, maintains firmness, retention of fruit skin color, and other sensory attributes (Khanzadi et al. 2023; Sharma et al. 2024). In this study, chitosan‐pectin‐based nanoemulsions showed significantly improved quality characteristics of the plum fruit compared to control samples.

The zeta potential values were recorded as −50.40, −88.60, and −109.10 mV for C_1_, C_2_, and C_3_, respectively. Higher absolute zeta potential values (beyond ±30 mV) usually mean that particles are electrostatically repelling each other well. This keeps particles from sticking together and improves the stability of colloids. The fact that the C_3_ nanoemulsion has a higher negative charge means a higher amount of CS‐pectin/EOs was present and applied on the surface, making the charge stronger. This means the particles do not stick together as much, and the emulsion is more stable. The results regarding the stability of the prepared nanoemulsion were in agreement with those of previously reported studies (Jayari et al. 2022; Keykhosravy et al. 2020; Mendes et al. 2020). Furthermore, carbonyl groups in EOs might be the major reason for stabilizing the negative charge on CS and maintaining the negative ζ‐potential.

### 3.2 FTIR Interaction Studies

**FIGURE 2:**
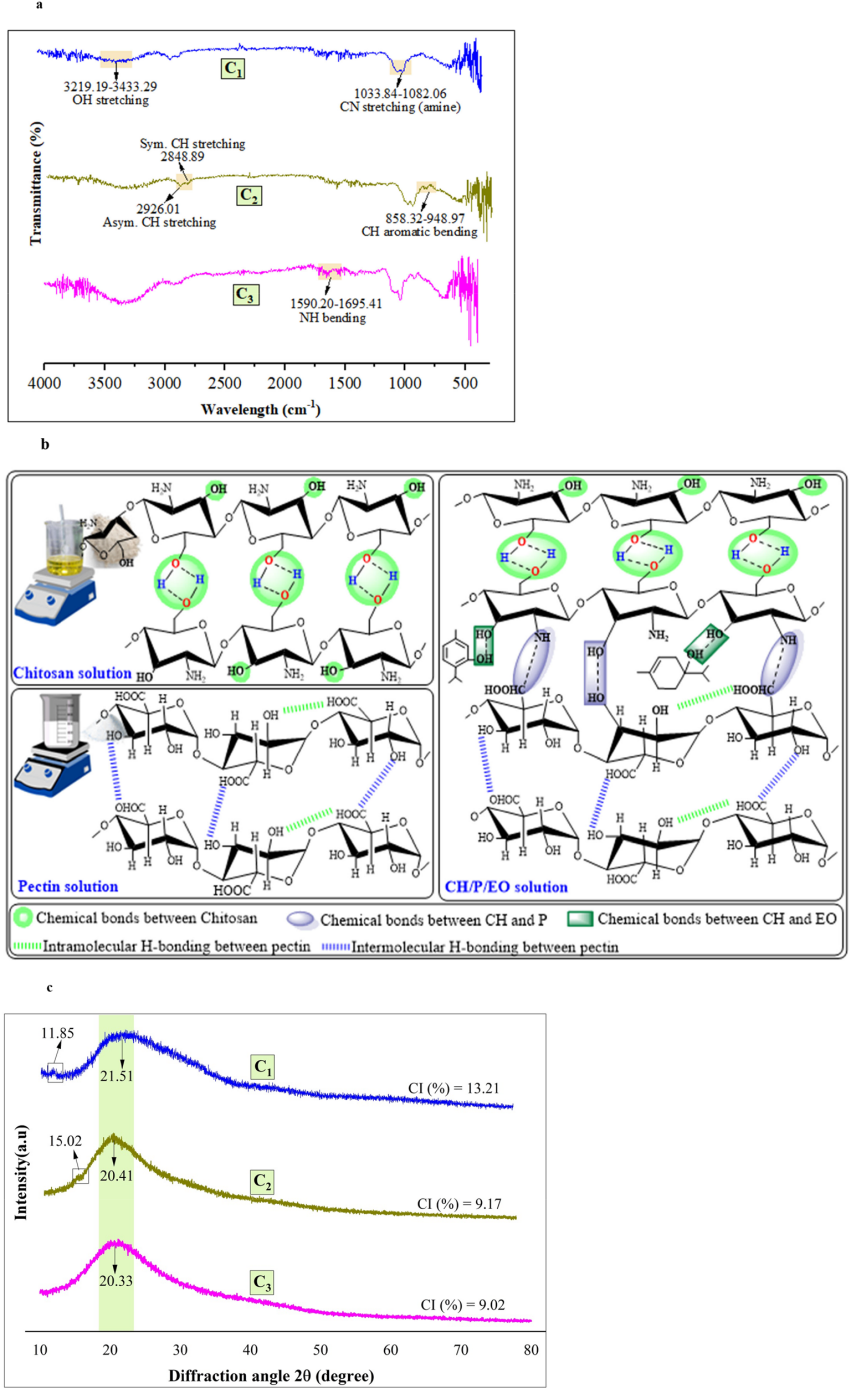
(a) FTIR analysis of chitosan, pectin, and prepared nanoemulsions. (b) The interaction of forces between the bioactive compounds of nanoemulsions. (c) XRD analysis of chitosan, pectin, and prepared nanoemulsions.

Figure 2a shows ATR‐FTIR spectra of CS‐pectin/EOs‐based nanoemulsions (C_1_, C_2_, and C_3_). The bands observed at 2848 and 2926 cm^−1^, corresponding to symmetric and asymmetric C‐H stretching, showed altered intensity when increasing concentrations of EO were incorporated into the nanoemulsions. The results indicated broader peaks with no new peak formation in prepared nanoemulsion samples, as previously reported in studies (Zhou et al. 2024).

A vibrational peak between 1590 and 1695 cm^−1^ was present in all samples, corresponding to N‐H bending vibrations, which are typical of amide II regions from CS (Caroni et al. 2021). The consistent presence of this peak confirms the contribution of CS's amino groups in the nanoemulsions. Besides, the absorption bands at 1033 and 1082 cm^−1^ are assigned to the polymer chain structure's CN stretching (amine) (Hashemi et al. 2025). A slightly higher peak intensity in C_3_ suggests a more vital intermolecular interaction in this nanoemulsion, possibly because of the higher CS concentration (1.5%). Additionally, a bending peak was observed between 858 and 948 cm^−1^, linked to C‐H bending, suggesting the aromatic components originating from the EOs (Akhter et al. 2024). Interestingly, the C‐H bending in the C_1_ sample is significantly smaller than in C_2_ and C_3_.

All samples showed a broad band of hydrogen‐bonded OH groups in the 3219–3433 cm^−1^ region. In coating C_3_, this peak appeared deeper, likely because of the higher concentration of polymer and EOs, suggesting a more extensive hydrogen bonding network (Hashemi et al. 2025). Figure 2b represents in detail the intramolecular and intermolecular hydrogen bonding. It has been observed that the band at the 3219–3433 cm^−1^ region in nanoemulsion coating C3 was broader than C_1_ and C_2_; this might explain the higher interaction of hydrogen bonding (Zhou et al. 2024). The –OH and –NH_2_ groups in CS and the –COOH group in pectin make the forces between molecules stronger (Caroni et al. 2021).

### 3.3 XRD Analysis

The XRD patterns presented in Figure 2c illustrate the crystallographic structure of the prepared nanoemulsions. The results have revealed insights into the interaction of different components of nanoemulsion on a molecular level, particularly in their crystalline or amorphous nature. The nanoemulsion C_1_, formulated with 1% CS and pectin combined with EOs, has displayed two distinct peaks at 11.85° and 21.51°, with a crystallinity index of 13.21%. These peaks indicate the presence of both an ordered crystalline region and a semi‐crystalline structure. Similarly, C_2_ exhibits two peaks at 15.02° and 20.41°, with a crystallinity index of 9.17%. The shift in the peak positions and a lower crystallinity index than in the C_1_ suggest a reduced crystallinity. In contrast, nanoemulsion (C_3_), formulated with higher biopolymer and EOs concentrations, showed a single broad peak at 20.33° with a crystallinity index of 9.02%. This may be due to the strong hydrogen bonding between EOs and CS, and similar phenomena were reported by Chen et al. (2025) and Zhang et al. (2021), who studied chitosan/pullulan and gellan gum/chitosan nanoemulsions containing EOs, respectively. The crystallinity index gradually decreased from 13.21% to 9.02%, the observed modifications may result from the incorporation of pectin/EOs into the crystal lattice of CS, which likely altered the inter‐planar distances, consistent with findings from FTIR analysis (Figure 2a). Similar results were reported by Akhter et al. (2024) and Azadi et al. (2023).

### 3.4 Morphology of Nanoemulsion by SEM

**FIGURE 3:**
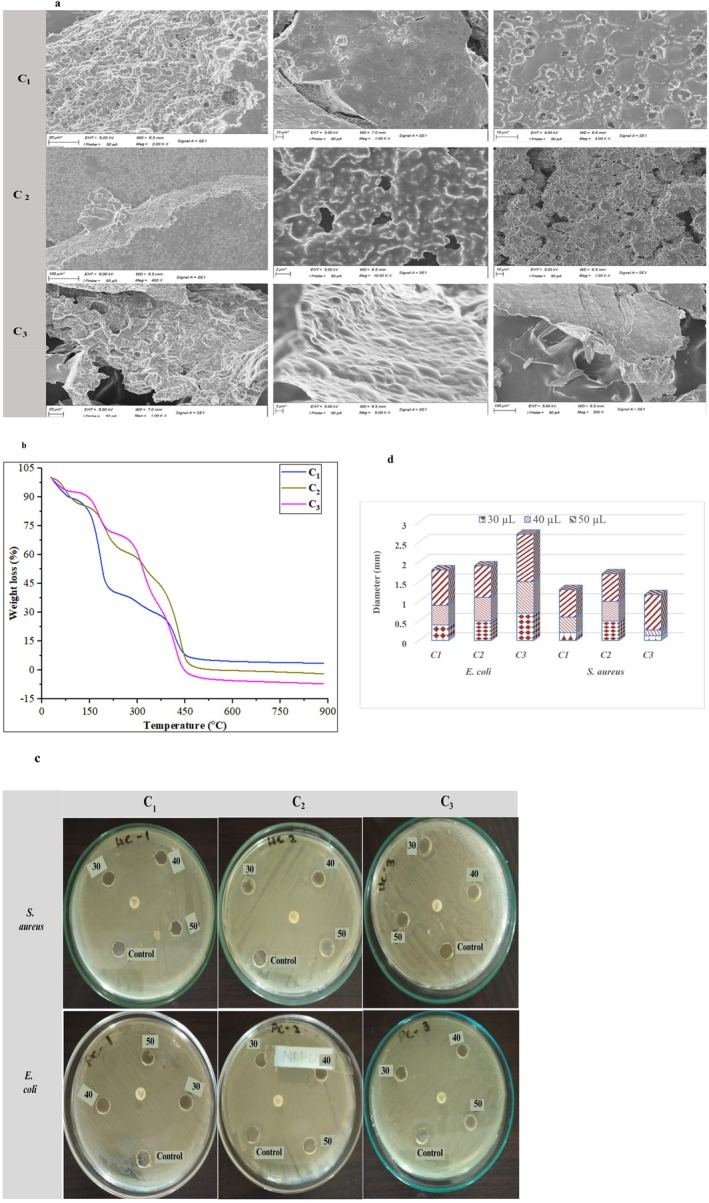
(a) Microstructure (SEM) analysis of prepared nanoemulsions at various magnifications. (b) TGA thermograms of chitosan‐pectin based nanoemulsions at different temperature scale. (c) The visual experimental results of bacterial growth against different amounts of prepared nanoemulsions, that is, C_1_, C_2_, and C_3_. (d) Reduction in the diameter (mm) inhibition zone (mm) of microbial organisms with different levels of prepared nanoemulsions. All values are presented as means ± SD (*n* =3).

The surface morphology of C_1_, C_2_, and C_3_ nanoemulsions was analyzed by SEM, and the samples revealed distinct differences (Figure 3a). The nanoemulsion C_1_ exhibited numerous pores and a uniform distribution of spherical or rounded small‐size globules, suggesting a porous and flexible structure. This flexibility could be due to the lower concentration of biopolymers and EOs, allowing for more surface deformation. In contrast, C_2_ demonstrated a more rigid and stiffer morphology with sharp edges and no visible deformation. This indicates enhanced structural integrity. Meanwhile, C_3_, with a higher concentration of biopolymers and EOs, showed a homogenous distribution and absence of observable pores, alongside a less flexible surface and signs of increased flocculation. As a result, a decrease in adhesion strength and spreading coefficient of the coating solution was observed, along with an increase in the cohesion coefficient, owing to the cross‐linking between polymers and EOs (Pirozzi et al. 2020). This observation is confirmed by DLS results, which showed a larger particle size of C_3_ than C_1_ and C_2_. The increase in particle size is likely due to the higher polymer concentration, promoting particle swelling and possible aggregation in the dispersion (Das et al. 2024). Similar SEM trends were reported by Hashemi et al. (2025), where the incorporation of EOs in nanoemulsions was found to influence their size and morphology.

### 3.5 Thermal Property of Nanoemulsion by TGA

The thermal stability of C_1_, C_2_, and C_3_ was evaluated using thermogravimetric analysis (TGA), as shown in Figure 3b. The thermal degradation process of biopolymer‐based materials generally follows three distinct phases (Dos Santos et al. 2023). Initially, moisture evaporates during the dehydration phase (up to 100°C) (Mao et al. 2023). It is followed by the thermal decomposition of saccharides and low molecular weight components around 200°C–300°C (Villegas et al. 2023). Lastly, the biopolymer matrix undergoes major structural breakdown above 300°C (Erceg et al. 2024; Rezaei et al. 2021). An initial weight loss below 100°C is attributed to all samples' moisture evaporation from the nanoemulsion. As can be seen, C_2_ and C_3_ exhibit thermal solid stability, with their primary decomposition beginning at 120°C and 145°C, respectively. The coating C_1_, on the other hand, is more thermosensitive, undergoing two distinct weight loss phases in its thermogram. The first phase, which accounts for 50% of the weight loss (between 85°C and 225°C), is linked to the degradation of low‐boiling‐point aromatic compounds of EOs. The second phase contributes to weight loss of 33% (from 225°C to 452°C), corresponding to the breakdown of high‐boiling‐point aromatic compounds such as thymol, carvacrol, and p‐Cymene (Abdalla et al. 2023).

The characteristic weight loss of CS, pectin, and EOs was no longer observed in C_3_ nanoemulsion, and the primary decomposition began at 150.5°C, indicating improved thermal stability as the concentration of biopolymers and EOs enhanced. This enhancement is likely due to the solution matrix's protective effect, which delays volatile compounds' thermal degradation. Previous studies have also shown that CS‐based nanoemulsions loaded with EOs can improve the thermal stability of volatile substances (Wang et al. 2023). The thermal stability of the nanoemulsion was significantly enhanced by increasing the amount of CS and pectin because both polymers created a synergistic effect, which made the structure of the nanoemulsion more stable. Wang et al. (2023) have documented that the stability of chitin nanofiber was improved when an extract peel of eggplant was added. The first weight loss happened between 25°C and 75°C, mainly related to water evaporation from the nanoemulsion. The second phase occurred between 150°C and 450°C; however, after 450°C, a negative weight loss of nanoemulsions C_2_ and C_3_ was observed, possibly because of weight gain in the presence of oxygen during combustion. Finally, a quasi‐constant weight loss was observed in nanoemulsion C_1_ at temperatures ≥ 450°C (Jamróz et al. 2022).

### 3.6 Antimicrobial Activities

The antimicrobial activity of the three prepared nanoemulsions (C_1_, C_2_, and C_3_) was evaluated by measuring the inhibitory zones formed against *Staphylococcus aureus* (Gram‐positive) and *Escherichia coli* , the common pathogens often associated with food spoilage (Luna‐Sosa et al. 2020; Rezaei et al. 2021). The C_1_ nanoemulsion demonstrated a reduced inhibition zone, lacking significant antibacterial activity against bacterial growth. However, the nanoemulsions (C_2_ and C_3_) with higher concentrations of biopolymers and EOs displayed markedly improved antibacterial properties compared to the coating of C_1_. Notably, as the volume of nanoemulsion coating increased, the inhibition zone against microorganisms was also enhanced. In Figure 3c, it demonstrates the visual experimental results of nanoemulsions against bacterial growth, and Figure 3d shows that nanoemulsion C_3_ at 50 μL volume inhibited *Escherichia coli* and *S. aureus* of 1.20 ± 0.06 and 0.90 ± 0.09 mm, respectively. The increase in antibacterial activity with higher volume and concentrations of polymers and EOs was statistically significant (*p* < 0.05), demonstrating the effectiveness of the oils in enhancing the antimicrobial performance of prepared nanoemulsions.

The inhibition of both Gram‐positive and Gram‐negative bacteria by the nanoemulsions containing EOs is mainly because of the polyphenolic compounds of oil (Wang et al. 2022). The positively charged amino group of chitosan interacts with the negatively charged bacterial cell membrane, leading to membrane disruption and cell death. Chitosan interferes with microbial replication. In the nanoemulsion form, the small droplet size enables better dispersion, increased surface coverage, and enhanced contact with microbes on the fruit surface (Maswanganye et al. 2025). These compounds, well known for their antimicrobial properties, disrupt bacterial cell diffusion, thereby preventing microbial growth and resulting in strong bacteriostatic effects. Compounds such as carnosic acid, rosmarinic acid, eugenol, and caffeic acid are responsible for the enhanced antibacterial activity (Bashir et al. 2023; Haneef et al. 2022). Thus, nanoemulsions containing EOs show strong potential for use as antibacterial food packaging material, which could effectively extend the shelf life of various food products, as in the current study, enhancing the shelf life of plum fruits. In our previous studies, nanoemulsions (containing carboxymethylcellulose/EOs and hydroxypropyl methylcellulose/beeswax/EOs) enhanced the antimicrobial activity and extended the shelf life of kiwifruit and sweet cherries, respectively (Iqbal, Haider, et al. 2024; Iqbal, Hussain, et al. 2024). The safe limit of yeast and mold count in Aloe‐pectin‐coated mango slices was found to be not greater than 5.0 log CFU/g (Khalil et al. 2022).

### 3.7 Physiological Attributes of Plums

#### 3.7.1 Visual Appearance and Weight Loss

**FIGURE 4:**
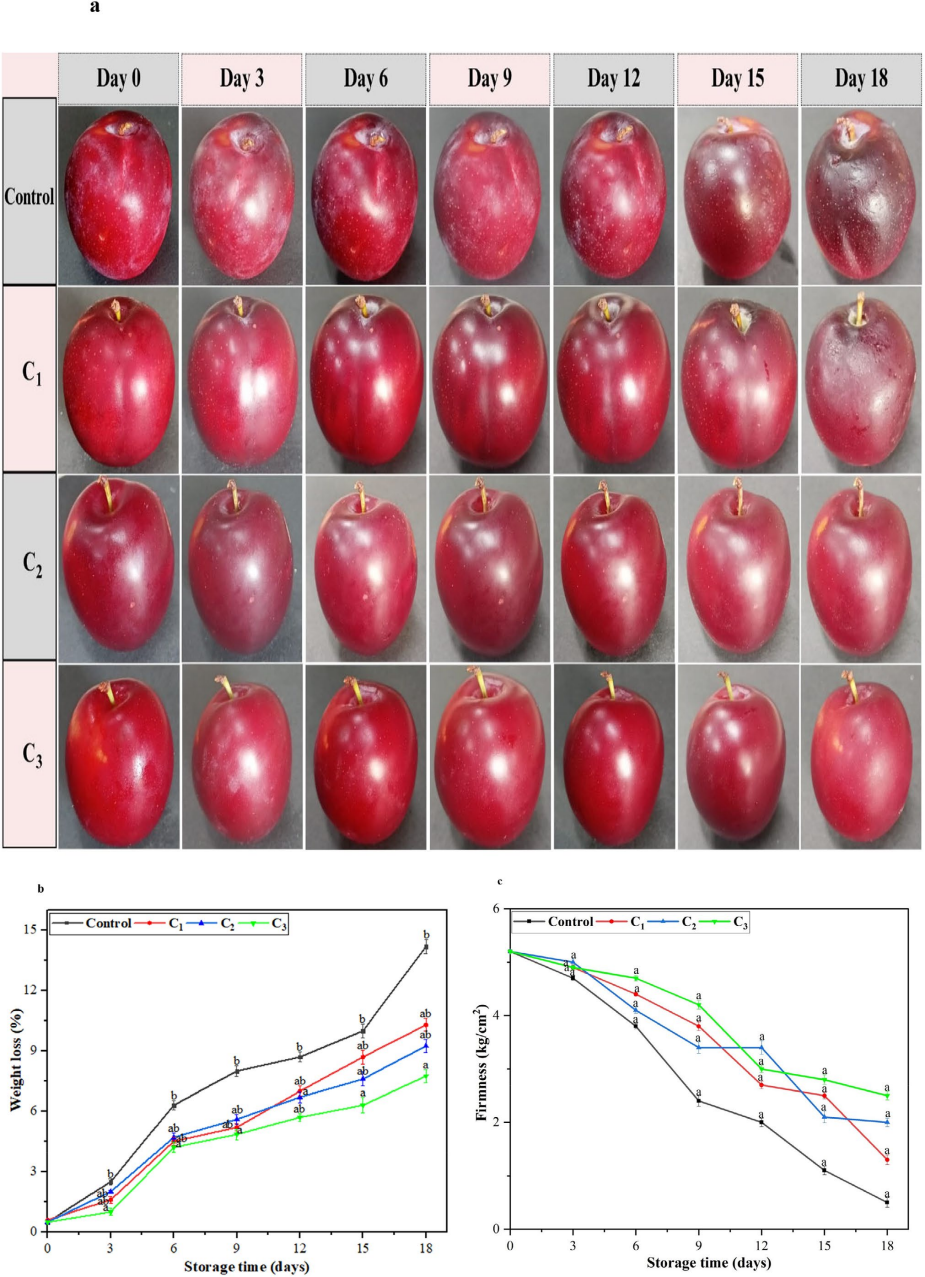
(a) Visual appearance of control and coated plum samples during the storage period of 18 days. (b) Line graph showing weight loss (%) of plum fruit samples (control and coated) during storage period. (c) Line graph representation of texture (firmness) of plum fruit samples of control and coated samples. All values are presented as means ± SD (*n* = 3). Small letters on the line graph indicate statistically significant differences between treatments on the corresponding storage day (*p* < 0.05).

The visual appearance of both control and coated plum samples during the storage period of 18th days showed a significant difference (Figure 4a). Control (C) plums exhibited more significant surface wrinkling, dulling of color, and a visibly degraded appearance as the storage period progressed. The surface deterioration became more prominent as spoilage intensified from the top of the plum fruit and eventually completely collapsed in shape by the 15th to 18th days. The C_1_‐coated plum fruits showed these changes but allowed noticeable wrinkling and fading of color by the 18th day, with partial loss of shape. On the other hand, the plum fruits coated with C_2_ and C_3_ maintained their shapes, smooth surfaces, and vibrant colors, which are crucial indicators of fruit freshness.

Figure 4b demonstrates the weight loss percentage in both coated and control plum samples during the storage period of 18 days. The results have revealed that C_1_ coating showed a non‐significant (*p* ≥ 0.05) increase in weight loss from day 6 to 9, that is, 4.5% to 5.22%. The results showed a significant (*p* < 0.05) decrease in weight loss of samples coated with edible coatings of C_1_, C_2_, and C_3_ compared to those samples placed in the control group. In the current research, after the 18th day, the weight loss of plum samples was 7.50% ± 0.21%, 9.25% ± 0.30%, and 10.30% ± 0.32% for edible coating of C_3_, C_2_, and C_1_, respectively.

The main factor for the reduction in weight loss of plum fruit during storage is the loss of water induced by respiration and transpiration processes (Gadallah et al. 2024). Weight loss in fruits and vegetables is generally associated with water loss from plant tissues, which occurs through transpiration and respiration processes, directly affecting cellular metabolism (Wang et al. 2023). Further water loss leads to fruit shriveling and tissue wilting. Postharvest storage conditions (temperature and relative humidity) are crucial in maintaining weight loss. The CS/pectin/EO nanoemulsions can help reduce transpiration and respiration by sealing the stomata and enhancing the mechanical strength of fruits (Muley and Singhal 2020).

#### 3.7.2 Firmness and Decay Incidence

There is a gradual decrease in firmness for coated and control plum samples during the storage period, as shown in Figure 4c, with a more intense decay observed for control fruits. The firmness of control plum samples was significantly (*p* < 0.05) decreased from 5.21 ± 0.01 to 0.50 ± 0.09 kg/cm^2^ at 18 days. The C_2_‐ and C_3_‐coated plum samples maintained their firmness levels between 2.10 ± 0.09 and 2.50 ± 0.08 kg/cm^2^. The results indicated no significant (*p* ≥ 0.05) difference in firmness retention between coated and control plum samples. In contrast, the decrease in firmness was substantially more significant in C_1_‐coated plum samples, which experienced more rapid softening compared to the C_1_‐ and C_3_‐coated plums.

The softening of fruit is generally driven by enzymatic reactions that are part of natural biochemical processes. These reactions degrade the cell structure, weaken cell wall composition, and deform intracellular materials. Some well‐known enzymes, such as wall hydrolases, polygalacturonase, and pectinase, are responsible. This enzymatic activity ultimately results in the softening and textural changes in fruits during postharvest storage, contributing to the quality deterioration of fruits (Aayush et al. 2022). The edible coatings may act as a barrier, preventing direct interaction between enzymes and their substrates, thereby delaying enzymatic activities that cause fruit softening. By reducing the impact of these enzymatic processes, the edible coatings effectively extend the firmness and shelf life of fruits during storage (Ishkeh et al. 2021; Panahirad et al. 2020). Previous studies (Khalil et al. 2022; More et al. 2022) have indicated that CS/pectin/EOs and other edible coatings have proven beneficial effects in maintaining the firmness of fruits.

**FIGURE 5:**
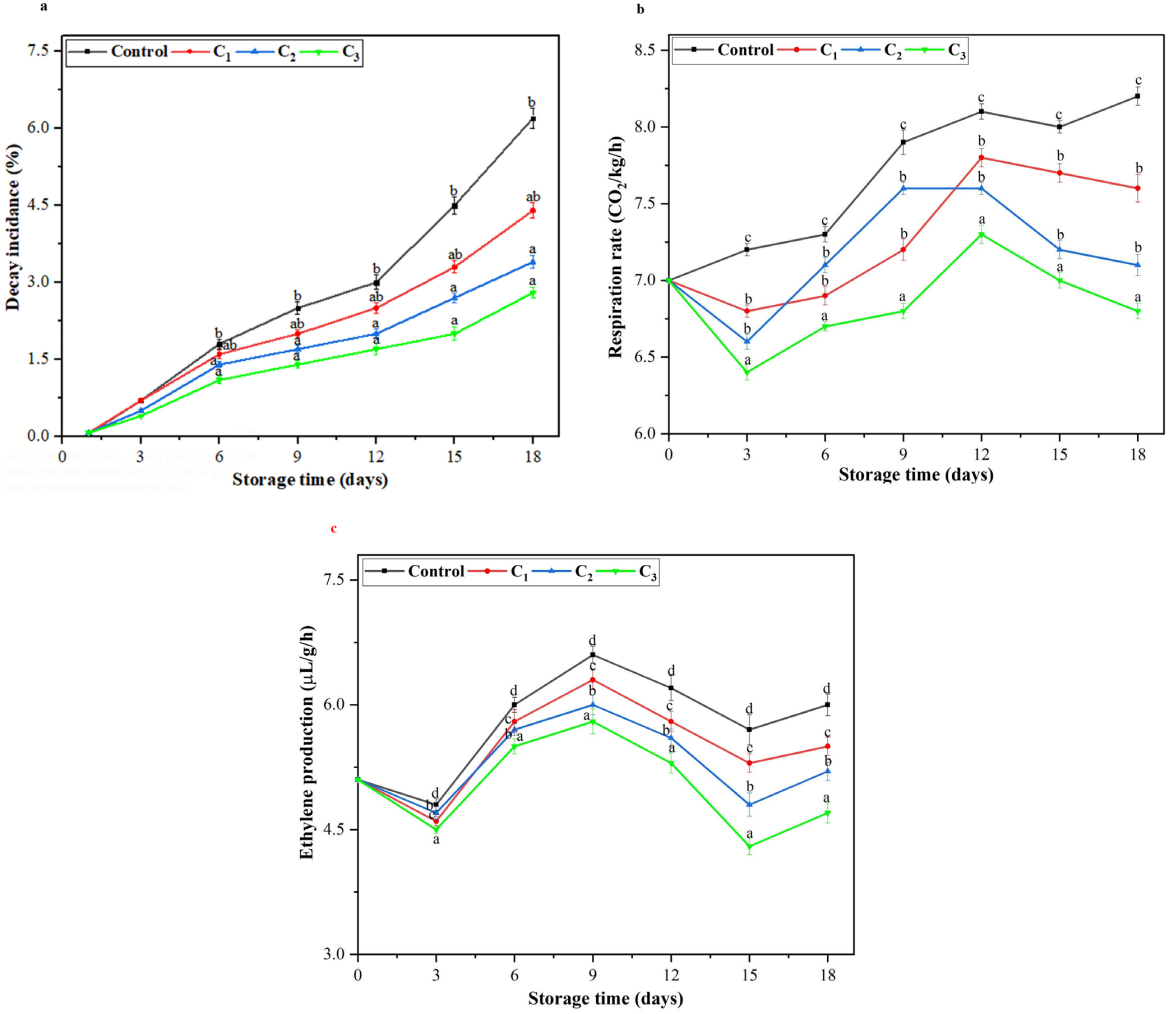
(a) Graphical representation of decay incidence (%) of plum fruit of control and the coated sample during the storage period. (b) Line graph of respiration rate (CO_2_/kg/h) of control and coated plum samples during the storage period. (c) Ethylene production (μL/g/h) representation of control and coated plum samples during the storage period. All values are presented as means ± SD (*n* = 3). Small letters on the line graph indicate statistically significant differences between treatments on the corresponding storage day (*p* < 0.05).

The physiological activities of fruits limit their shelf life after harvesting. Figure 5a illustrates the effect of edible coatings on the percentage of decay in plums during storage. Both control and coated plum samples exhibited an increase in percentage decay over the storage period. However, the edible coatings significantly reduced the percentage of decay compared to the control plums. Initially, the control plum samples had a decay percentage of 0.10%. However, by the 18th day of storage, 6.23% of the control plum samples were infected with decay. In contrast, the coated samples showed significantly lower decay, ranging from 0.12% to 4.40% during the same storage period. Among the edible coating formulations, C_3_ effectively reduced the percentage decay to just 2.82%. The enhanced performance of this emulsion coating in preventing spoilage can be attributed to the improved antimicrobial activities of EOs, which inhibit mold growth by creating a protective surface on the fruit. Several studies have demonstrated that biopolymer‐based nanoemulsion blended with different types of EOs effectively decreases the decay rate. Chen et al. (2025) reported that strawberries coated with chitosan/pullulan edible coating combined with thyme essential oil exhibited significantly lower decay rate.

#### 3.7.3 Respiration Rate and Ethylene Production

The RRs with the application of nanoemulsion on the plum fruit samples are depicted in Figure 5b. The results have indicated that the RR increased in control plum samples during storage, and the control group showed the highest RR. Statistical analysis showed a significant difference (*p* < 0.05) between the RR of control and coated plum samples after 12 days. The results showed a gradual increase in RR for all coated samples over the whole storage period, irrespective of the samples coated with nanoemulsion C_3_. It was observed that the plum group coated with C_1_ (1% CH/PEOs) was inefficient in reducing the RR. However, plum samples coated with C_3_ (1.5 CH/P and 2:2:1% EOs) demonstrated greater effectiveness in lowering the levels of RR. The highest RRs were observed in the control samples at 8.34 ± 0.07 CO_2_/kg/h, whereas the lowest RR, 6.40 ± 0.04 CO_2_/kg/h, was observed in the coated plum samples. These findings align with previous research on plums and other fruits, confirming the effectiveness of edible coatings in slowing down RRs (Fawole et al. 2020; Sivakumar et al. 2021).

The formation of an ionic‐crosslinked network, resulting from the aggregated structure of CS, pectin, and EOs, is believed to regulate and modify the internal atmosphere of fruit tissues efficiently. This structural arrangement could be crucial in controlling gas exchange within the fruits. Additionally, with semipermeable membrane properties, CS‐based coatings can slow down fruit ripening by altering the concentration of CO_2_ and O_2_ in the fruit's interphase. On the basis of this fact, biodegradable polymer‐based coatings have the most effective impact on reducing the RR of fruits and vegetables (Rana et al. 2024).

As observed in Figure 5c, ethylene production was increased for control plum samples and suppressed for coated plum samples during the storage period of 18 days. The ethylene levels were increased from a baseline of 5.11 ± 0.01 μL/g/h on day 0 to an average concentration of 7.60 ± 0.15 μL/g/h for the control plum sample on the 12th day. Plum samples coated with C_3_ had the lowest level (5.32 ± 0.12 μL/g/h on the 12th day) compared with other treatments (C_1_ and C_2_), which had intermediate levels (7 ± 0.10 and 6 ± 0.08 μL/g/h), respectively. Interestingly, on the 15th day, the ethylene rate was again suppressed for the control and coated plum samples. However, the levels of ethylene were increased again on the last day of storage for control and C_1_‐coated plums from 5.70 ± 0.18 to 6.50 ± 0.13 and 5 ± 0.11 to 5.50 ± 0.09 μL/g/h, respectively. In contrast, the levels of ethylene of plum samples coated with C_2_ and C_3_ during the 18th day storage period.

It is well‐recognized that ethylene production is dependent on oxygen levels, and it is obvious that a reduction in internal oxygen typically leads to a lower level of ethylene. This may suggest that the edible coatings applied on the surface of plum fruit samples could control the oxygen level, consequently suppressing the level of ethylene (Sivakumar et al. 2021).

#### 3.7.4 Color

**FIGURE 6:**
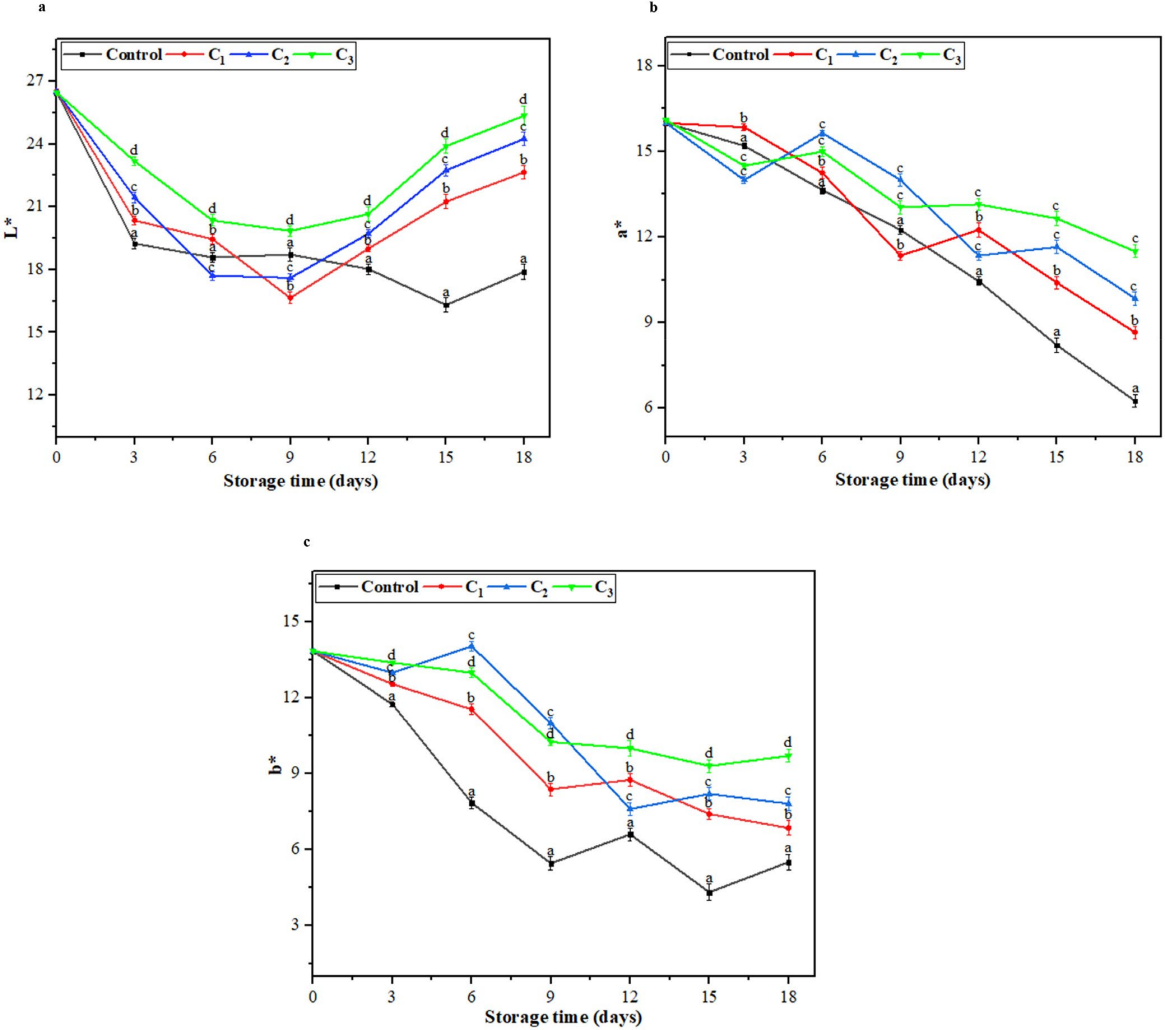
(a–c): Color (L*, a*, and b*) analysis of control and coated plum samples with during storage period. All values are presented as means ± SD (*n* = 3). Small letters on the line graph indicate statistically significant differences between treatments on the corresponding storage day (*p* < 0.05).

The color change in fruits during the storage period indicates a loss of freshness, which directly impacts consumer appeal and marketability of the product. An increase in the lightness (L*) of plums was observed as the concentration of nanoemulsion in the edible coatings increased (Figure 6a). However, over the storage period, a decrease in L* value was observed in all samples of plums, reflecting the natural browning process directly linked with fruits' degradation. The control group (control plum samples) showed a significant increase in browning with a decrease in the L* value from 26.45 on the initial day to 17.91 by the 18th day of storage. On the other hand, C_1_, C_2_, and C_3_ nanoemulsion coatings effectively reduced the extent of browning in the treated plums. Specifically, in C_3_‐coated plums, the L* values decreased from 26.45 on 0 days to 25.35 on the last day of storage, suggesting that higher concentrations of the nanoemulsion helped to maintain the lightness and delay browning during the storage period of plums. This could be due to the protective barrier provided by coatings, which lowers moisture loss and oxidation, key contributors to browning.

In the present study, there is a continual and significant decrease in a* values (green to redness) of control samples (from 16 ± 0.08 to 6.20 ± 0.21) compared to the coated samples (*p* < 0.05), which exhibited almost similar behavior (Figure 6b). However, C_3_‐coated plums showed the lowest decrease from the 0 days (16.11 ± 0.05) to the 18th (11.50 ± 0.22) day of storage. Although visual color differences were subtle, instrumental measurements confirmed that coated plums with CS‐pectin/EO‐based formulation achieved lower red color intensity than control plum samples. Edible coatings likely play a crucial role in slowing down the degradation of chlorophyll and the accumulation of carotenoids, thereby delaying the ripening process. The change in a* parameter is linked to the ripening process of fruits, where chlorophyll breaks down in the chloroplast while pigments (carotenoid and anthocyanins) increase, and this transition enhances the redness of fruits (Prakash et al. 2020).

Similarly, the b* value (yellowness) of plums increased with higher concentrations of CS‐pectin/EO‐based nanoemulsion in prepared edible coatings (Figure 6c). The reduction in yellowness was also better controlled in coated samples of plums. On the last day of the shelf life of plums, the b* value of the control plum samples had decreased to 5.50 ± 0.31 from 13.85 ± 0.01. In contrast, C_3_‐coated samples exhibited a smaller decrease among the coated plums, with the b* value reduced by only 9.72 ± 0.24 from 13.82 ± 0.01 and showing better retention of the yellowness. Similar findings have been reported in previous literature, where different formulated edible coated plums have been shown to significantly improve preservation by reducing color degradation (Fawole et al. 2020; Sivakumar et al. 2021).

### 3.8 Biochemical Properties of Plum

#### 3.8.1 TA and TSS

**FIGURE 7:**
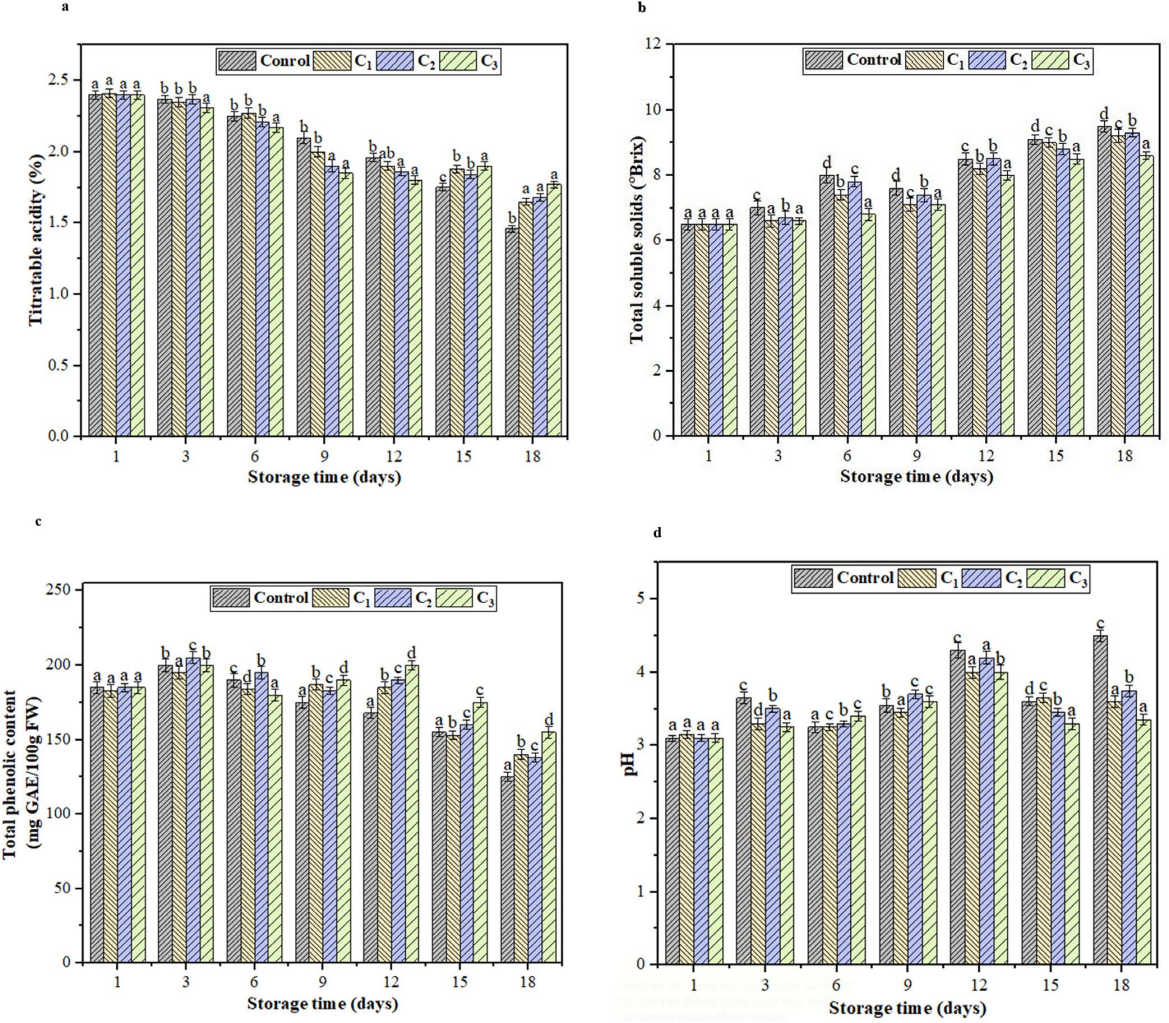
(a) Titratable acidity (TA) of control and coated plum samples at different storage periods. (b) Total soluble solids (TSS) brix of control and coated plum samples during different storage period. (c) Total phenolic contents (mg GAE/100 g FW) of control and coated plum samples at different storage periods. (d) The pH of control and coated plum samples at different storage periods. All values are presented as means ± SD (*n* = 3). Small letters on the bar chart indicate statistically significant differences between treatments on the corresponding storage day (*p* < 0.05).

The impact of various coatings on the acidity of the plum fruit samples is presented in Figure 7a. Throughout the storage period, both control and coated plum samples exhibited a gradual decline (*p* < 0.05) in TA. Initially, the plum fruits had no noticeable differences in acidity levels. However, significant differences were observed during 18 days of storage. At the beginning of the storage period, the acidity levels ranged from 2.41% to 1.46% for control plum samples, from 2.41% to 1.65% for C_1_, from 2.42% to 1.68% for C_2_, and from 2.40% to 1.78% for C_3_‐coated plum samples. This could be attributed to consuming organic acids during respiration, with the coated plum samples showing low levels of decrease in TA. Edible coatings may inhibit the respiration of plums. They can slow down the consumption of acid, thus effectively slowing the decline rate of titratable acidity and extending the shelf life of plums (Miranda et al. 2022).

Generally, it is accepted that a higher total soluble solid content indicates greater fruit ripeness. Moreover, minimal fluctuations in TSS levels during storage indicate better fruit preservation (Asgarian et al. 2023). As depicted in Figure 7b, the TSS content of the control samples increases as the storage period progresses. In contrast, coated samples show a slower rise, with the C_3_ coating maintaining a more stable and nearly constant TSS level. The TSS content of the control plum samples was initially 6.52° ± 0.01° and grew to 12° ± 0.22° Brix on the final day of storage. In contrast, coated plum samples (C_3_) have shown a gradual increase in TSS levels and documented a value of 6.4° ± 0.02° Brix on the 1st day, which was found to be 8.20° ± 0.11° Brix at the 18th day. The results of the current study prove the influential role of edible coatings as selective barriers to carbon dioxide and oxygen gases, consequently modifying the internal atmosphere of plums, followed by slowing down the RR. Hao et al. (2024) observed similar results for plums applied with a composite of buckwheat starch and xanthan gum edible coating.

#### 3.8.2 TPC and pH

The TPC of the plum fruits was significantly influenced by the C_3_ nanoemulsion treatment, with the treated plum fruits consistently showing the highest levels of TPC by the 12th day of storage (Figure 7c). The highest total phenolic content (200 ± 3.21 mg GAE/100 g FW) was observed in samples treated with C_3_ edible coatings. The lowest content (125 ± 2.32 mg GAE/100 g FW) was recorded in control samples after the 18th day of storage. This increase in C_3_‐coated samples might be due to the production of phenolic compounds and other compounds with antioxidant properties generated through the physiological processes of the fruits (Al‐Dairi et al. 2021). The phenolic content of both control and coated plums gradually declined after 12 days of storage. It could be due to the fruit aging and increased respiration, leading to the breakdown of cellular structures. In contrast, the coated samples exhibited a smaller decline in TPC, which can be attributed to the coating materials' barrier properties and reducing the oxidative degradation of phenolic compounds (Haneef et al. 2022).

Figure 7, illustrates the change in pH of plums during the storage period. The data revealed a statistically significant (*p* < 0.05) increase in pH over time, with notable differences between control and coated plum samples. By the last day of storage, the control plums showed the highest pH increase, reaching 4.52 ± 0.09. On the other hand, the coated plums also rose; however, they remained significantly lower than the control. The pH value indicates a fruit sample's acidity or basic nature, which affects its taste and flavor during postharvest storage (Nogueira et al. 2021). The C_3_ nanoemulsion proved more effective than C1 and C_2_ nanoemulsions in maintaining the pH levels closer to the initial value. The pH results of this study are consistent with the previous reports (Kocira et al. 2021).

### 3.9 Sensory Evaluation

**FIGURE 8:**
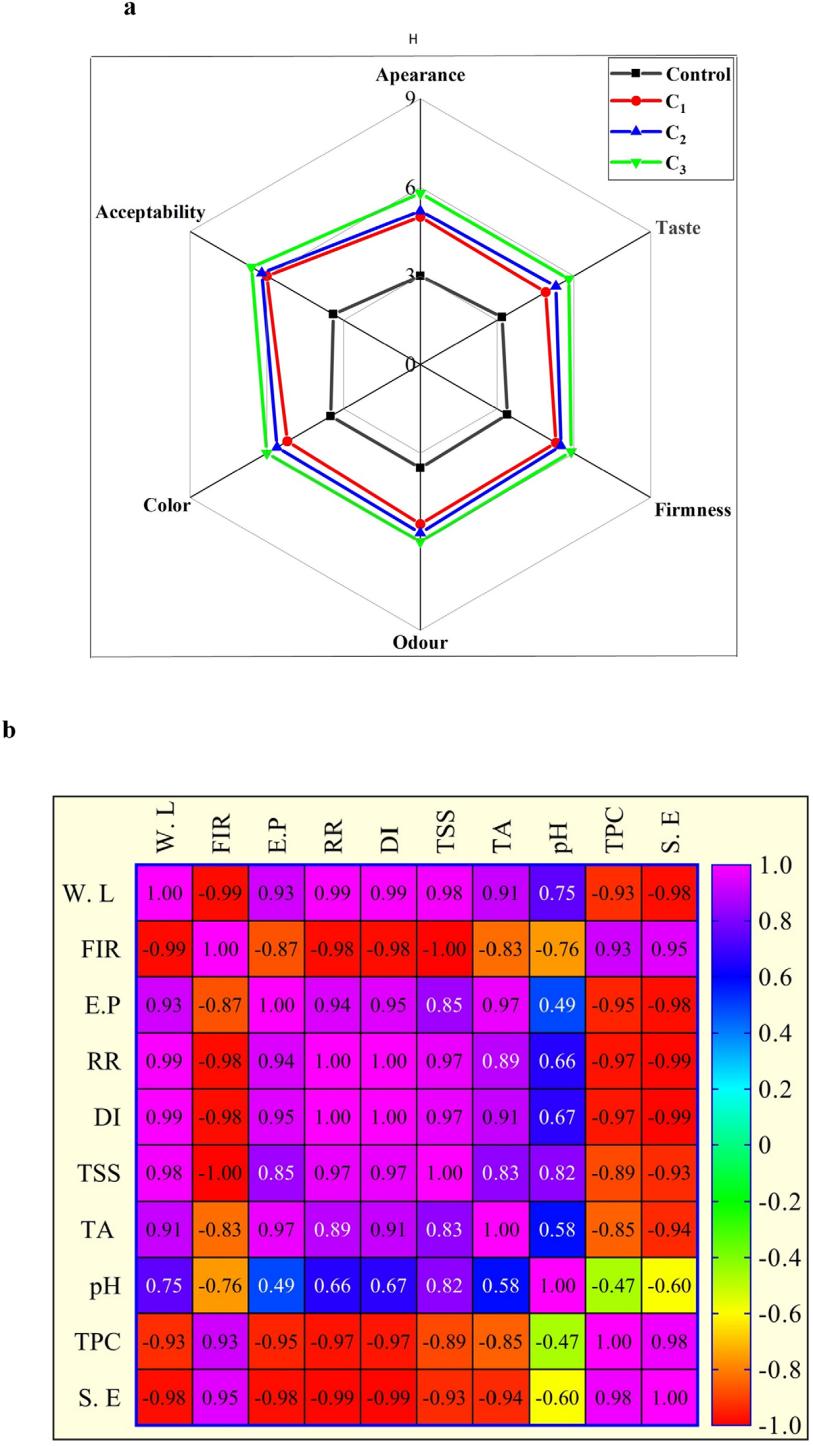
(a) Sensory evaluation of control and coated plum samples at different storage periods. (b) Heatmap correlation analysis of different parameters during different storage periods. Positive correlation coefficients range from 0 to 1, where 0 indicates no correlation, and 1 indicates a perfect positive correlation. Negative correlation coefficients range from 0 to −1, where 0 indicates no correlation, and −1 indicates a perfect negative correlation. DI = decay incidence, E.P = ethylene production, FIR = firmness, RR = respiration rate, S.E = sensory evaluation, TA = titratable acidity, TSS = total soluble solids, TPC = total phenolics contents, W.L = weight loss.

The sensory characteristics of all the samples were examined at the end of the storage period to assess their overall consumer appeal and acceptability (Figure 8a). Significant variations were evaluated across all parameters, including appearance, taste, firmness, odor, color, and overall acceptability (OAA). The results have indicated that no noticeable changes were detected on the first day in evaluated parameters; however, on the final day of the storage, the panelists reported significant differences in all the attributes mentioned above of control and coated plum samples. The control plum samples scored 3, whereas the coated plums scored 4.90, 5.20, and 5.80 on the hedonic scale for C_1_, C_2_, and C_3_ nanoemulsions, respectively. This trend suggests that the coatings enhanced the visual appearance of plums, making them more attractive to consumers. The control group of plums received a rating of 3.20 for taste. On the other hand, coated plum samples have scored higher values at 4.90 (C_1_), 5.30 (C_2_), and 5.80 with C_3_ coating.

Regarding firmness, the control group's rating was 3.40, whereas C_1_, C_2_, and C_3_ coated samples showed improvements with scores of 5.30, 5.50, and 5.90, respectively. The enhanced firmness contributes to the positive perception of the fruit's texture. Similar trends were observed for the odor and color ratings, with the control group scoring lower (3.50) than the coated plum samples, which received significantly higher scores, culminating in a perfect score of 6 for the color of samples coated with C_3_. The overall acceptability scores reflected a clear preference for the coated plum samples, with the control group at 3.40, whereas C_1_, C_2_, and C_3_ scored a ranking of 6, 6.20, and 6.60, respectively. This indicates that the coatings enhanced the individual sensory attributes and improved consumer acceptance. It has been documented that a covering of CS resulted in lower levels of RRs and gas permeability control on fruits and vegetables.

### 3.10 Correlation Analysis

Figure 8b indicates the analysis of the Pearson coefficient to conduct a correlation‐based analysis, which revealed substantial negative (in red) and positive (in blue) connections between all parameters during the preservation of food. The results have indicated that weight loss (*r* = 1.00), ethylene production (*r* = 0.93), RR (*r* = 0.99), total soluble solids (*r* = 0.98), titratable acidity (0.91), decay incidence (*r* = 0.99), and pH (*r* = 0.75) levels showed strong positive correlation. However, firmness (*r* = −0.99), TPC (*r* = −0.93), and sensory evaluation (*r* = −0.98) have shown a strong negative correlation. The results indicated that a positive correlation existed between the parameters that determine the decay and browning of the fruit.

## 4 Conclusion

This research effectively formulated and analyzed a CS pectin‐based edible coating infused with essential oils (cinnamon, peppermint, and rosemary). The implementation of this coating demonstrated favorable outcomes in preserving the quality of the stored plum fruit, especially with nanoemulsion C_3_, specifically by diminishing weight loss, pH levels, TSS, TA, ethylene production, RR, and decay index, while sustaining color index, sensory attributes, and firmness of the stored fruits. The antibacterial properties of the coating helped maintain the firmness and other physical characteristics of the plum samples. Furthermore, it substantially inhibited the activity of enzymes associated with fruit softening and ripening. Moreover, the coating resulted in a decrease in bacterial proliferation during storage. A recent study has utilized pectin and CS in conjunction with essential oils to make nanoemulsions aimed at prolonging shelf life and enhancing postharvest management of plum fruit and potentially other fresh agricultural products.

## Author Contributions

**Shahzad Zafar Iqbal:** conceptualization (equal), funding acquisition (equal), project administration (equal), resources (equal), supervision (equal), visualization (equal), writing – review and editing (equal). **Muhammad Waseem:** formal analysis (equal), resources (equal), validation (equal), writing – original draft (equal). **Farah Naz:** software (equal), visualization (equal), writing – review and editing (equal). **Munawar Iqbal:** validation (equal), writing – review and editing (equal). **Muhammad Adnan Ayub:** resources (equal), writing – review and editing (equal). **Osama A. Mohammed:** visualization (equal), writing – review and editing (equal). **Ahmad Faizal Abdull Razis:** project administration (equal), resources (equal), writing – review and editing (equal).

## Disclosure

AI and AI‐Assisted Technologies in Writing: The AI tool, that is, Quilbot, was used solely for the improvement of the English language, and the authors fully understand and take responsibility for the wording of the manuscript.

## Conflicts of Interest

The authors declare no conflicts of interest.

## Data Availability

Data will be made available on request.
